# The effectiveness of hepa filtered rooms plus fluconazol prophylaxis for preventation of invasive fungal diseases in allogenic stem cell transplant patients

**DOI:** 10.1186/2047-2994-4-S1-P274

**Published:** 2015-06-16

**Authors:** C Altay Kurkcuoglu, G Metan, L Kaynar, F Elmali, E Alp, M Cetin

**Affiliations:** 1 Infection Control Committee, Erciyes University Hospital, Kayseri, Turkey; 2 Infectious Diseases and Clinical Microbiology, Hacettepe University Faculty of Medicine, Ankara, Turkey; 3 Infectious Diseases and Clinical Microbiology, Erciyes University Faculty of Medicine, Kayseri, Turkey; 4 Hematology, Erciyes University Faculty of Medicine, Kayseri, Turkey; 5 Biostatisitics, Erciyes University Faculty of Medicine, Kayseri, Turkey

## Introduction

In our hematopoieteic stem cell transplantation (HSCT) center, preventation of invasive fungal diseases (IFDs) for the patients who did not experience IFDs previously is based on performing allogeneic SCT in rooms with “High efficiency particulate air” (HEPA) filters and administration of fluconazole prophylaxis.

## Objectives

We aimed to evaluate our prophylaxis policy to ensure if preventive measures are working.

## Methods

Erciyes University Hospital is a 1300-bed tertiary centre with a 38-bed HSCT center. We retrospectively reviewed records of 146 ASCT episodes between January 2012 and December 2014 to detect the patients with IFDs before engraftment. Patients who experince IFDs before ASCT were excluded from the study. As fluconazole has no activity against *Aspergillus spp*, an early diagnostic policy for invasive aspergillosis (IA) was guided by twice weekly *Aspergillus* Galactomannan antigen detection from the day of neutropenia until engraftment and radiological interventions when clinically required. The European Organization for Research and Treatment of Cancer and the Mycoses Study Group criteria were used to categorize the patients as having proven, probable, or possible IA. IA is accepted as nosocomial if patient was hospitalized more than seven days and no history of previous IFDs in the last six months.

## Results

The 105 out of 146 ASCT episodes were from matched related donor, 10 were from matched unrelated donor, and 31 were haploidentical ASCT. Possible IA was diagnosed in 10 patients, probable IA was diagnosed in 3 patients, proven IA in 1 patient, and fungemia was detected in 4 patients. *Candida* mucositis was diagnosed in 7 patients. The crude mortality rate in three months after ASCT was 10.4% (4 patients with IFDs *vs* 11 patients without IFDs) in 144 ASCT patients who were followed by our center.

## Conclusion

ASCT in rooms with HEPA filters with positive pressure, combined with fluconazole prophylaxis prevented IFDs in 82.8% of the 146 ASCT episodes in pre-engraftment period.

## Disclosure of interest

C. Altay Kurkcuoglu: None declared, G. Metan Grant/Research support from: Associates of Cape Cod, Conflict with: Member of Advisory board for Pifizer, Gilead, Astellas, L. Kaynar: None declared, F. Elmali: None declared, E. Alp: None declared, M. Cetin: None declared.

